# Diffusion of soluble organic substrates in aerobic granular sludge: Effect of molecular weight

**DOI:** 10.1016/j.wroa.2022.100148

**Published:** 2022-07-02

**Authors:** Lenno van den Berg, Sara Toja Ortega, Mark C.M. van Loosdrecht, Merle K. de Kreuk

**Affiliations:** a Department of Water Management, Delft University of Technology, the Netherlands; b Department of Biotechnology, Delft University of Technology, the Netherlands

**Keywords:** Diffusion, Biofilms, ESEM, Molecular weight, Aerobic granular sludge

## Abstract

Aerobic granular sludge (AGS) is an advanced biofilm-based technology for wastewater treatment. Diffusion of substrates into the granules is a key aspect of this technology. Domestic wastewater contains soluble organic substrates of different sizes that could potentially diffuse into the granules. In this study, the relation between the molecular weight of a substrate and its diffusion coefficient within the granule was studied with model substrates (polyethylene glycols (PEGs) with a molecular weight between 62 and 10 000 Da). The diffusion coefficients of the model substrates within granules from a full-scale installation were measured with the ‘transient uptake of a non-reactive solute’ method. The diffusion coefficients in the granules were not significantly different from the diffusion coefficients in water, at least up to 4000 Da molecular weight. This indicates that these PEGs were not obstructed by the granule matrix. The 10 kDa PEG behaved differently from the lighter PEGs, as it could not penetrate the entire granule. Furthermore, the granule structure was characterized with Environmental Scanning Electron Microscopy (ESEM). The granules displayed an open structure with large macropores and semi-solid regions, which contained microbial cells. The diffusion results suggest that most diffusing molecules were unobstructed in the macropores and barely obstructed in the semi-solid regions. Only the diffusion of the 10 kDa PEG seemed to be hindered by the semi-solid regions, but not by the macropores. Lastly, the apparent molecular weight distribution of domestic wastewater soluble COD was determined with ultrafiltration membranes of 100, 10, and 1 kDa molecular weight cut-off. The influent fractionation revealed that a large part (61–69%) of the influent soluble COD was lighter than 1 kDa. As molecules lighter than 1 kDa diffuse easily, the majority of the influent soluble COD can be considered as diffusible COD. These findings provide new insight into the availability of influent COD for granular sludge.

## Highlights

• Solutes with a molecular weight up to 4 kDa diffused rapidly into aerobic granules.
• The granules had a porous structure with large macropores and dense cell clusters.
• A solute of 10 kDa only penetrated the macropores, not the cell clusters.
• More than 60% of the soluble COD in three Dutch wastewaters was ≤ 1 kDa.
• Most soluble COD can diffuse easily into aerobic granules.

## Graphical abstract

**Figure:**
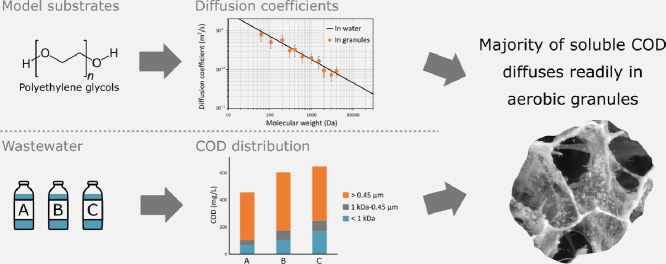

## 1 Introduction

Aerobic granular sludge (AGS) is a recent innovation for the treatment of wastewater (Pronk et al., 2015). The technology consists of microorganisms that produce extracellular polymeric substances (EPS) to form granular biofilms or granules. Due to the limited penetration of oxygen into the granule, different redox conditions can exist throughout the granule. Therefore, nutrient removal processes such as nitrification, denitrification, and phosphorus uptake, can occur simultaneously in a single reactor (Kishida et al., 2009; Wei et al., 2014). Sedimentation tanks are no longer needed due to the high settling velocity of the aerobic granules (Bengtsson et al., 2018). As a result, aerobic granular sludge technology requires less energy and land area compared to conventional activated sludge installations (de Bruin, de Kreuk, van der Roest, Uijterlinde, and van Loosdrecht, 2004; Pronk et al., 2015).

Most AGS research has been carried out with lab-scale systems that were fed with small and soluble substrates, like volatile fatty acids (VFAs) or glucose (Adav et al., 2008; de Kreuk, Heijnen, and van Loosdrecht, 2005; de Sousa Rollemberg et al., 2018). These substrates can diffuse readily into the granules, even into deeper zones (Pronk et al., 2015). The diffusivity of the substrates has a major impact on the overall performance of granular sludge reactors (Layer et al., 2019). A deep penetration of substrates will contribute to the formation of stable granules and to a high denitrification activity (Layer et al., 2020; Zheng et al., 2006). At the same time, often only a small fraction of real wastewater consists of small and soluble substrates. The concentration of VFAs in domestic wastewater is generally less than 10% of the influent chemical oxygen demand (COD) (Henze et al., 1995). The other soluble substrates in the wastewater can account for 10–20% of the influent COD (Henze et al., 1995). The exact nature and size of this non-VFA soluble COD fraction is poorly understood. Soluble COD can be as small as acetate, but also as large as a colloid (up to 0.45 µm). As a result, the diffusion behaviour of the soluble COD in the granules is poorly understood as well.

Diffusion in different biofilms has been researched extensively in the 1980s and the 1990s (Stewart, 1998). However, there are several limitations that hamper our knowledge on diffusion in biofilms and granules. Firstly, most studies focused on small substrates, like oxygen, ammonium, acetate, and glucose (Chiu et al., 2006; Liu et al., 2009; Stewart, 1998). Only a handful of studies have measured diffusion coefficients of molecules that are heavier than 300 Da (Bryers and Drummond, 1998; Peulen and Wilkinson, 2011; Takenaka et al., 2009; Thurnheer et al., 2003). It is therefore not clear how the size of a solute affects its diffusion behaviour. Secondly, different studies often yield diffusion coefficients that vary greatly (Stewart, 1998). For example, the mean relative effective diffusion coefficient (D_biofilm_/D_aq_) for solutes with a molecular weight between 44 and 342 Da was reported as 0.29 ± 0.24 (Stewart, 1998). This large variability is partially the result of differences in biofilm density between studies (Horn and Morgenroth, 2006), but it is not clear if other factors play a role as well. Lastly, our knowledge on diffusion in biofilms is limited due to the inherent inaccuracy of the methods that are commonly used to measure diffusion coefficients (van den Berg, van Loosdrecht, and de Kreuk, 2021). Overall, the understanding of diffusion in biofilms and granules is very limited, especially regarding heavier molecules (> 300 Da).

The objectives of this study were (1) to determine the effect of the molecular weight of a molecule on its diffusion coefficient in aerobic granules, (2) to determine the pore structure of the granules, and (3) to determine the distribution of soluble COD in different wastewaters. The diffusion coefficients of different polyethylene glycols (PEGs), uncharged model substrates with a molecular weight between 62 and 10 000 Da, were measured with the ‘transient uptake of a non-reactive solute’ method. The diffusion behaviour was related to the granule structure observed by ESEM. The distribution of soluble COD in domestic wastewater samples was determined through ultrafiltration with a 100, 10, and 1 kDa molecular weight cut-off.

## 2 Materials and methods

### 2.1 Granule source

Aerobic granules were harvested from the full-scale Nereda® plant in Utrecht, The Netherlands. The plant treats domestic wastewater with a COD of 649 ± 173 mg/L, a BOD_5_ of 300 ± 95 mg/L, and a TSS of 297 ± 95 mg/L (all average values with standard deviation during the 3 month sampling period). The average sludge loading rate was 0.05 kg COD/kg DS/d. The solids retention time was 20–50 days. The reactor was operated with biological phosphate removal. The sampled granules were sieved to retain only granules with size of 2.0–2.5 mm and afterward washed repeatedly to remove any non-granular material. Washing of the granules was done in three steps: First, the granules were suspended in tap water. Second, the granules were allowed to settle for a short time. Third, the liquid on top of the granules was decanted. These three steps were repeated around 5–10 times. This way, all material that did not settle well was removed from the granule sample. The granules were stored in tap water at 4 °C for a maximum of 2 weeks.

### 2.2 Diffusion experiments

Experiments to determine solute diffusion coefficients in aerobic granules were carried out with the ‘transient uptake of a non-reactive solute’ method (Westrin and Zacchi, 1991). The experiments were conducted with a working volume of 300 mL in a 500 mL jacketed glass vessel. Adequate mixing was provided by an orbital shaker (Heathrow Scientific Digital Orbital Shaker, 19.2 mm orbit diameter, 170 rpm). The orbital shaker was chosen to limit granule breakage from excessive shear (de Graaff, van Dijk, van Loosdrecht, and Pronk, 2018). The temperature was controlled at 4.0 ± 0.1 °C to limit biological activity.

Two solutions were prepared for the experiment: one with the granules and one with a specific molecule. For the granule solution, a certain amount of granules was added to a volumetric flask of 200 mL volume. The amount of the granules was chosen to obtain an α-value (the ratio of water volume, V_W_, over granule volume, V_G_) of roughly 4. The flask was then filled to 200 mL with tap water. For the specific molecule solution, a known mass of a specific solute was added to 100 mL of tap water. The solutions were pre-chilled and added to the jacketed glass vessel quickly to start the experiment.

Samples were taken at irregular intervals to best capture the non-linear bulk liquid concentration profile over time. For each experiment, 25 samples of 0.5 mL were taken through modified pipette tips. Regular plastic 1 mL pipette tips were covered in a stainless steel woven mesh with a mesh size of 100 µm to prevent the granules from clogging the pipette tip. The volume lost through sampling was immediately replaced by an equal amount of a solution with a concentration of the expected final solute value, as described in Nguyen and Luong (1986). At the end of each experiment, the temperature was increased to 20 °C and the granule volume was determined with the modified Dextran Blue method (van den Berg, Pronk, van Loosdrecht, and de Kreuk, 2021). Finally, total suspended solids and volatile suspended solids of the granules were determined according to Standard Methods (APHA, 2005).

Polyethyleneglycols (PEGs) of different molecular weight were used as solute in the diffusion experiment. Reagent-grade PEGs with average molecular weights of 62, 106, 200, 300, 400, 600, 1000, 1500, 2000, 4000, and 10 000 Da were obtained from Merck. The PEGs with molecular weight of 62 and 106 Da were monodisperse, while the other PEGs had a reported polydispersity index between 1.12 and 1.30. The initial PEG concentration in each experiment was approximately 1 mg/mL, with the exact value determined for each experiment separately. The diffusion coefficients of the different PEGs were taken from Waggoner et al. (1995).

### 2.3 Data analysis

The diffusion coefficients (D_e_) of the molecules in the granules were derived from the concentration change in the bulk liquid. If granules are initially free of substrate and the entire granule is accessible for diffusion, the decrease in bulk liquid concentration can be described by the following equation (Crank, 1975):

(1) $$\frac{C_{B}\left(t\right)}{C_{B}\left(t_{0}\right)}=\frac{1}{1+\alpha }\left(\alpha +\sum_{n=1}^{\infty }\frac{6\alpha \left(1+\alpha \right)\exp-\frac{D_{e}q_{n}^{2}t}{R^{2}}}{9+9\alpha +q_{n}^{2}\alpha^{2}}\right)$$

Here, C_B_(t) is the bulk liquid concentration at time t, R is the granule radius, α is the ratio of liquid volume over granule volume, and the q_n_’s are the non-zero positive roots of

(2) $$\tan q_{n}=\frac{3q_{n}}{3+\alpha q_{n}^{2}}$$

The granule volume, water volume (= total experimental volume – granule volume), and granule radius (see Section 2.6) were determined experimentally. The initial solute concentration could not always be measured accurately in the experimental setup, since diffusion of the solute into the granule started before the liquid volume concentration was completely homogeneous. Instead, the initial concentration was calculated from the weight of solute added and the water volume in the experiment.

Non-linear least squares fitting of the diffusion model (Eq. (1)) to the experimental bulk liquid concentration data was used to find the best approximation for the diffusion coefficient. In order to ensure that the global optimum was found, rather than a local optimum, random initial values were used repeatedly. For each experiment, 500 random initial values were used that varied between 1 · 10^−13^ and 2 · 10^−9^ m^2^ *s* ^−^ ^1^. The precision of the diffusion coefficient was estimated according to the procedure of Alper and Gelb (1990). With this procedure, the uncertainty in the granule radius, granule volume, and PEG concentrations was propagated to the diffusion coefficient. One thousand Monte Carlo simulations were used to approximate the standard deviation of the fitted diffusion coefficient. The obtained diffusion coefficients at 4 °C were all converted to corresponding values at 25 °C based on Einstein (1905):

(3) $$D_{25}=D_{4}\frac{T_{25}}{T_{4}}\frac{\mu_{4}}{\mu_{25}}$$

Here, D is the diffusion coefficient (m^2^/s), T is the absolute temperature (K), and µ is the dynamic viscosity of water.

### 2.4 Environmental scanning electron microscope analysis

Several granules were examined by environmental scanning electron microscopy (ESEM) to relate diffusion with the granule pore structure. ESEM does not require desiccation or coating of the granules. Therefore, the granules can be imaged without any pre-processing and in a fully hydrated state. A Quanta FEG 650 (FEI Company, USA) was used with a gaseous secondary electron detector and a Peltier cooling stage set at 0.5 °C. The granules were imaged at pressures between 3 and 7 mbar, which corresponds to a relative humidity of 50–100%. Several granules were sliced into two halves with a scalpel to examine the inner structure of the granules with ESEM as well. As reference sample, alginate beads were included in the analysis. The beads were prepared by dissolving sodium alginate (sigma) in demineralised water to a 2% w/v solution and dripping this solution into a 2.5% w/v CaCl_2_ solution. The alginate beads were allowed to harden for 30 min and subsequently stored in tap water.

### 2.5 Influent characterization

Influent samples from three wastewater treatment plants (WWTP) were analysed to determine the molecular weight distribution of the soluble organic fraction. The influent samples originated from three WWTPs in The Netherlands, all treating domestic wastewater: from Utrecht WWTP in Utrecht (480 000 million population equivalents (p.e.)), from Harnaschpolder WWTP in Den Hoorn (1.3 million p.e.), and from Bath WWTP in Rilland-Bath (485 000 p.e.). These three WWTPs were selected because of the different sewer systems: Utrecht WWTP is fed from a relatively short gravity sewer system, Harnaschpolder WWTP is fed from both short and longer pressure mains, and Bath WWTP is fed from a long pressure main. Around 44% of the influent to Bath WWTP was industrial wastewater, originating from petrochemical, chemical, and waste processing facilities. Flow-proportional composite samples from a 24 h period were collected after the influent screening (6 mm). The samples were collected in the months October, November, and January, during dry weather flow conditions. Immediately after collection, the samples were stored at 4 °C, for a maximum of 3 h prior to analysis. After a brief settling period, the samples were filtered in an Amicon ultrafiltration cell (Merck Millipore). The filtration was carried out serially with a 0.45 µm filter (Durapore PVDF, Merck Millipore) and ultrafiltration membranes of 100, 10, and 1 kDa nominal molecular weight cut-off (Ultracell regenerated cellulose, Merck Millipore). The membranes were treated according to the manufacturer's instructions. The filtration was performed at 20 °C and with a pressure of 2 bar from nitrogen gas.

### 2.6 Analytical methods

Chemical oxygen demand (COD), phosphate (PO_4_-P), and ammonium (NH_4_–N) in the (filtered) wastewater samples were measured in triplicate with Hach Lange test kits. The COD measurements was based on the dichromate method. Total suspended solids and volatile suspended solids in the wastewater samples were determined in triplicate according to Standard Methods (APHA, 2005). VFA in the wastewater were analysed with high performance liquid chromatography (HPLC; Prominence, Shimadzu, Japan), equipped with an ion exchange column (Aminex HPX-87H, Bio-rad) and a UV index detector (SPD-20A, Shimadzu, Japan). Sulphuric acid in ultrapure water (5 mM) was used as eluent. The individual VFA concentrations of acetate, propionate, and butyrate were converted to COD and lumped together to yield the total VFA concentration in mg COD/L. The concentration of PEG samples was measured with the same HPLC system, equipped with two size exclusion chromatography columns in series (SUPREMA 5 µm 30 Å, PSS GmbH, Germany) and a refractive index detector (RID-20A, Shimadzu, Japan). Ultrapure water was used as eluent at a flow rate of 0.8 mL/min. The size distribution of the granule samples was determined with a digital microscope (Keyence VHX-700F). The microscope images were processed with ImageJ software to obtain a granule size distribution and an average aspect ratio (Schneider et al., 2012).

## 3 Results

### 3.1 Diffusion coefficients in AGS

**Fig. 1:**
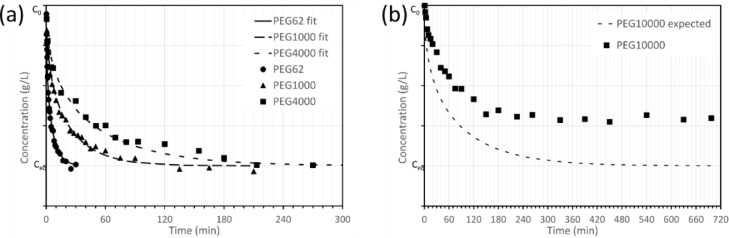
(a) Concentration profile of the diffusion experiments with PEG molecules of molecular weight 62, 1000, and 4000 Da, including a line for a fitted diffusion model. (b) Concentration profile of the diffusion experiment with the 10 kDa PEG, including the expected concentration profile. The expected profile was calculated based on Eq. (1) and the diffusion coefficient of the 10 kDa PEG in water.

The diffusion coefficients of different PEG molecules within aerobic granules were determined with the ‘transient uptake of a non-reactive solute’ method. Each experiment yielded a bulk liquid concentration profile over time, an average granule radius, a granule volume, and the liquid volume. Examples of the concentration profiles from the transient uptake experiments are given in Fig. 1. Most concentration profiles followed a non-linear decrease from the initial concentration (C_0_) to the equilibrium concentration (C_eq_). There was no significant consumption of the solute during the experiments, since consumption of the solute would result in a final concentration lower than the expected equilibrium concentration. A detailed overview of the individual diffusion experiments is given in Table A.1 and Figure A.1.

**Fig. 2:**
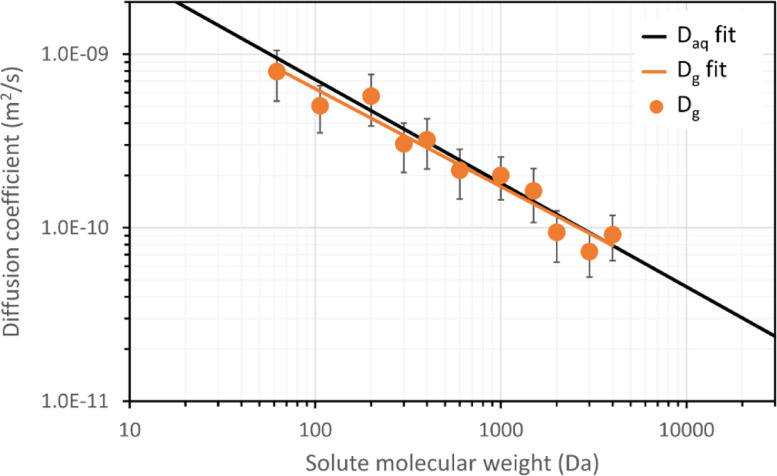
Diffusion coefficients of PEGs in aerobic granules as function of PEG molecular weight, at 25 °C. The orange solid line indicates the fitted values for the diffusion coefficients in the granules. The black solid line indicates the fitted values for the diffusion coefficients of PEGs in water (Waggoner et al., 1995).

For the PEG molecules between 62 and 4000 Da, the relative diffusion coefficient (D_e_/D_aq_) in the granules was between 0.73 and 1.22. The diffusion coefficients of the PEGs in the granule displayed large variability, with standard deviations ranging from 28 to 34%. These large standard deviations are probably the cause of the relative diffusion coefficient larger than 1 (for PEG200, 400, 1000, 1500, and 4000). The diffusivity of PEGs in the granule decreased logarithmically with the molecular weight of the PEGs, similar to the diffusivity of PEGs in water (see Fig. 2). The relation between diffusivity and molecular weight is given by:

(4) logD=alogMW+b

Here D is the diffusion coefficient, MW is the molecular weight, coefficient a is the slope, and coefficient b is the intercept. For PEG diffusion in water, the slope is −0.597 ± 0.020 and the intercept is −7.951 ± 0.077 (Waggoner et al., 1995). For PEG diffusion in the granules, the slope and intercept found in this study are −0.564 ± 0.044 and −8.070 ± 0.126, respectively. A *t*-test revealed that the slope of granules and water were not significantly different from each other, t(18) = 0.68, *p* = .51. The same applied for the intercept, t(18) = 0.81, *p* = .43. Thus, the granule matrix had no significant effect on the diffusion of PEG of all the tested molecular weights, from 62 to 4000 Da.

For almost all diffusion experiments, the equilibrium concentration was equal to the expected equilibrium concentration. This indicates that the concentration in the bulk liquid was equal to the final concentration in the whole granule. There was one exception, namely the 10 kDa PEG molecule (see Fig. 1B). The concentration in this experiment stabilized at a higher level than expected. As a result, no diffusion coefficient could be extracted from this experiment. The diffusion model that is used to determine the diffusion coefficient (see Eq. (1)) requires that the expected equilibrium is reached. The high equilibrium concentration in this experiment indicates that not all granule volume is accessible for this 10 kDa molecule. A mass balance of PEG at the final equilibrium (Eq. (5)) showed that only 65% of the granule volume was accessible for this molecule mass.

(5) $$C_{\mathrm{eq}}\left(f_{\mathrm{acce}\mathrm{sible}}V_{g}+V_{w}\right)=C_{0}V_{w}$$

Here, f_accesible_ is the fraction of the granule volume that is accessible for a molecule.

### 3.2 Characterization of the granules

**Fig. 3:**
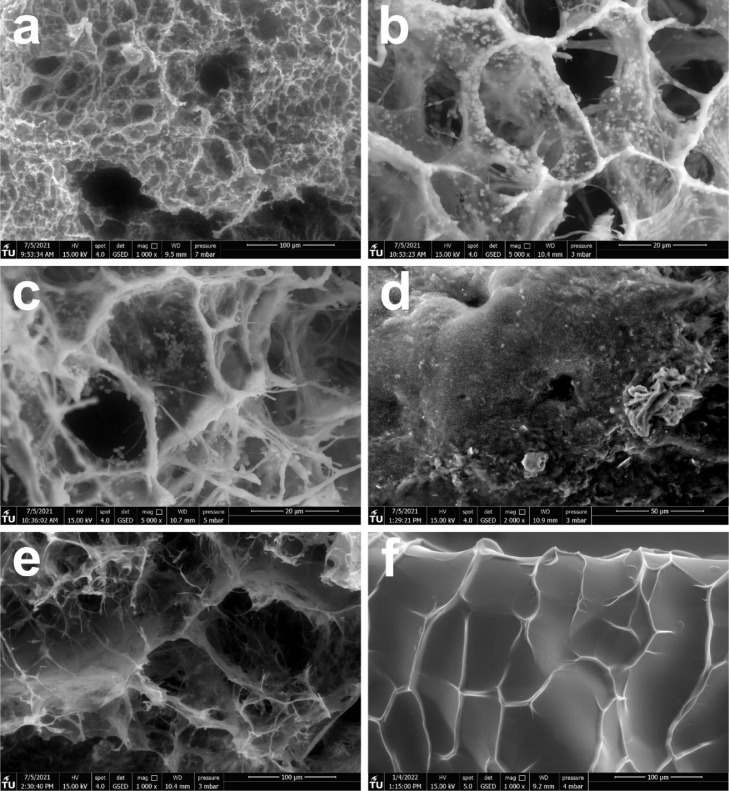
ESEM images of the surface and inner sections of aerobic granules from a full-scale WWTP. (A to C) Images that show the heterogeneous surface of the granules: The surface is characterised by several ‘holes’ and dense polymer strands. Individual cells are distinguishable as well, as white blobs. (D) A homogeneous section of the granule surface. In this image, the cells are clustered close together. (E) The core of a sliced granule. Large voids and a very open structure are visible. (F) The surface of an alginate bead, showing a dense surface without distinguishable pores. Note that the scales differ between images.

The hydrated structure of the granules was visualized with ESEM, to gain insight into the medium through which the PEG molecules diffused. An overview of the collected ESEM images is given in Fig. 3. The images reveal a complex granule architecture. The majority of the granule surface was heterogeneous and many large voids with a diameter of 10–20 µm were visible (Fig. 3A-C). It is unclear exactly how far these voids penetrate into the granules. Still, the lack of signal from these voids (the black colour in the ESEM images) suggests that they are not shallow. The granule surface also showed thick strands that form a connected network. These fibril-like strands were of similar diameter as the microbial cells which are visible as well: around 0.5–1 µm (Fig. 3A-C). The length of the polymer strands was in the range of 10–30 µm. Some parts of the granule surface were much less heterogeneous and displayed dense clusters of cells underneath a smooth surface (Fig. 3D). However, only a minority of the granule surface had this more homogeneous nature.

The inside of the granules was even more open than the granule surface. Fig. 3E shows in the centre of the sliced granule, the structure was almost completely open and large voids were visible. The structure of the surface of alginate beads, which were included as reference material, was very different from that of the aerobic granules (Fig. 3F). The surface of the beads showed vein-like structures, which could be the result of shrinking during the preparation of the beads. However, aside from these structures, the surface was completely smooth and no pores could be identified.

### 3.3 Influent characterization

Influent wastewater was collected from three WWTPs. The sewer systems feeding the WWTPs differed in terms of hydraulic retention time and sewer type. The wastewaters were all fractionated to reveal the apparent molecular weight distribution of soluble COD. As can be seen in Table 1, the three wastewaters showed similar characteristics. The majority of the COD was present in the particulate form (62–77%). Around 15–26% of the total COD was present in the smallest size fraction, which was below 1 kDa in size. Only a minor fraction (11–12%) of the influent COD was present in the fractions from 1 kDa to 0.45 µm. This means that the majority (61–69%) of the soluble COD in all three wastewaters was lighter than 1 kDa. The VFAs were only a minor fraction of the soluble COD (17–21%).

**Table 1:** COD fractionation and other characteristics of three influent wastewater samples. The COD fractions were determined through serial filtration. All values are reported in mg/L, including standard deviation. The VFA concentration is given in mgCOD/L.

		Utrecht	Harnaschpolder	Bath
COD fractionation				
	Total	605 ± 9	456 ± 20	649 ± 44
	Soluble (< 0.45 µm)	175 ± 1	105 ± 1	248 ± 1
	100 kDa-0.45 µm	11 ± 2	21.6 ± 0.2	23 ± 4
	10–100 kDa	42 ± 2	9.5 ± 0.8	10 ± 4
	1–10 kDa	17 ± 2	19.8 ± 1.1	45 ± 2
	< 1 kDa	106 ± 1	67.2 ± 0.8	170 ± 1
Additional parameters				
	TSS	260 ± 1	221 ± 8	268 ± 12
	TN	61.7 ± 1.4	49.0 ± 0.6	59.0 ± 1.7
	NH_4__—_N	45.9 ± 0.2	33.8 ± 0.5	42.2 ± 0.6
	TP	8.0 ± 0.2	6.22 ± 0.03	10.97 ± 0.06
	PO4-P	4.6 ± 0.1	3.39 ± 0.03	7.47 ± 0.15
	VFA	30.8 ± 0.7	17.7 ± 0.4	36.3 ± 0.2

## 4 Discussion

### 4.1 Diffusion coefficients of PEG molecules

In the past, many researchers have studied diffusion in biofilms. There appeared to be a relation between the molecular weight of a solute and its relative diffusivity. In biofilms, the reported relative diffusivity of small solutes (< 44 Da) was 0.46, while that of large solutes (44–342 Da) was 0.39 (Stewart, 1998). The relative diffusivities found in this study were much higher, between 0.73 and 1.22. Furthermore, there was no discernible effect of the solute molecular weight on the relative diffusivity for molecules between 62 and 4000 Da. This is surprising, as the molecular weight range used in this study is much greater than the molecular weight range included in the review of Stewart (1998).

One possible explanation for the discrepancy relates to the method used in this study. The ‘transient uptake of a non-reactive solute’ method has been used previously to measure diffusion coefficients in hydrogel beads and biofilms (Chresand et al., 1988; Fan, Leyva‐Ramos, Wisecarver, & Zehner, 1990; Pu and Yang, 1988). The limitations of this method has been described previously, highlighting the low precision as major issue (van den Berg, van Loosdrecht, et al., 2021; Westrin and Zacchi, 1991). This low precision was observed here as well, but it cannot explain the large difference between our results and the results described in literature. Alternatively, the diffusion coefficients in this study could be overestimated due to systematic errors or biases. However, the effect of most systematic errors for this method is that the diffusion coefficient is underestimated (van den Berg, van Loosdrecht, et al., 2021). There are only two systematic errors that lead to an overestimation: deactivation of the biomass and a non-spherical granule shape. We do not expect any bias from deactivation, as we did not use deactivating chemicals. The granule shape could have an influence, as ellipsoidal granules have a higher surface-to-volume ratio than perfectly spherical granules. As a result, the concentration in the liquid decreases faster and the diffusion coefficient is overestimated if granules are assumed to be spherical. However, the average aspect ratio of the granules was only 1.44. Our previous research has shown that this corresponds to an overestimation of the diffusion of only 10% (van den Berg, van Loosdrecht, et al., 2021). Thus, it is not likely that our findings significantly overestimate the diffusion coefficient. In fact, the diffusion coefficient could even be slightly underestimated due to the rough surface of the granule, the granule size distribution, or the mass transfer boundary layer (van den Berg, van Loosdrecht, et al., 2021).

Another explanation for the difference between our study and previous studies relates to the methods and types of biofilm used in the previous studies. A thorough analysis of the studies included in the review of Stewart (1998) revealed several issues (see Table A.1 for full details). In total, there were 21 studies on diffusion of larger molecules (with molecular weight of 44–342 Da). From these 21 studies, 12 used the ‘steady-state reaction’ method, which is very imprecise and inaccurate (van den Berg, van Loosdrecht, et al., 2021). Furthermore, 5 out of the remaining 9 studies were performed on biofilms with a density greater than 150 g/L. It is not surprising that the diffusion coefficient in such a high-density biofilm is much lower than in aerobic granules with a density of 90 g/L (Horn and Morgenroth, 2006). The remaining 4 studies were performed with sludge flocs (3) or without considering biomass activity (1). As a result, all studies on larger molecules that are included in Stewart (1998) have clear limitations and a direct comparison with our findings is futile.

There are several studies not included in the review of Stewart (1998) that focused on molecules in the kDa range. These studies are commonly carried out with fluorescent dextran molecules and fluorescence correlation spectroscopy or fluorescence recovery after photobleaching. These techniques measure diffusion coefficients in specific locations in a biofilm instead of the average diffusion coefficients measured in this study. Several studies of kDa-sized molecules report high relative diffusivities for molecules between 3 and 10 kDa in biofilms (Takenaka et al., 2009; Zhang et al., 2011) and alginate beads (Favre et al., 2001; Puguan et al., 2015). For example, Peulen and Wilkinson (2011) found a relative diffusivity of 60–80% for a 3 kDa and a 10 kDa dextran in a Pseudomonas fluorescens biofilm. Others reported relative lower diffusivities, ranging from 0.01 to 0.23 for molecules with a molecular weight between 3 and 10 kDa (Bryers and Drummond, 1998; Lawrence et al., 1994; Marcotte et al., 2004; Thurnheer et al., 2003). The reason behind this wide range in reported values is unclear, but it could be due to differences in biofilm density or structure.

Overall, we believe our results are representative for diffusion in aerobic granules, despite the large standard deviations. The main result of the experiments are not the exact diffusion coefficients, but rather the relation between the molecular weight and diffusion coefficient. These results have been collected with the same method and the same type of biofilm. Thus, even if the absolute diffusion coefficients would not be reliable, the overall trend would remain the same. As it turns out, the granule matrix has no significant effect on PEG molecules of 62–4000 Da. This conclusion is in contrast with existing literature and provides a new perspective on diffusion, specifically in aerobic granules. Our study has focused on the molecular weight with neutrally charged molecules. Future research should investigate the effect of charge and hydrophobicity on diffusion in aerobic granules.

### 4.2 Granule structure

The limited effect of the solute molecular weight on diffusion raised the question why the granule matrix did not provide more obstruction to the larger molecules. To address this question, we investigated the structure and porosity of the granule matrix with ESEM imaging. In contrast to conventional electron microscopy, ESEM does not require dehydration or fixation the granule. Therefore, ESEM is an appropriate technique to visualize the EPS matrix in its natural, hydrated state (Priester et al., 2007).

The ESEM images of the granules (see Fig. 3) revealed that the granules were very heterogeneous with arguably two distinct phases: a liquid phase and a semi-solid phase. The liquid phase is present in large macropores that are visible on the granule surface and in the granule interior. The semi-solid phase in the granule was present in between the macropores, and many microorganisms were embedded within this semi-solid phase. The presence of macropores or channels and cell clusters is an established observation in biofilm literature (Bryers and Drummond, 1998; Marcotte et al., 2004; Peulen and Wilkinson, 2011; Picioreanu et al., 2016; Sankaran et al., 2019; Takenaka et al., 2009).

Generally, porosity is used as a parameter to compare the volume of the macropores with the volume of the cell clusters. However, porosity in biofilms is an ill-defined concept, since both the liquid phase in the macropores, as well as the semi-solid phase mostly consists of water (Lewandowski, 2000). Diffusion occurs both in the macropores and in the semi-solid phase. The size of micropores in the semi-solid phase could not be determined with ESEM imaging. However, alginate hydrogels have a typical pore size of 5–20 nm (Boontheekul et al., 2005; Leal-Egaña et al., 2011; Simpliciano et al., 2013; Smidsrød and Skja, 1990). The size of the micropores in the granules could be of a similar order of magnitude, since alginate gels are similar to the EPS from AGS (Felz et al., 2020; Lin et al., 2010; Schambeck et al., 2020).

The hydrodynamic radius of the PEGs used in this study ranged from 0.15 to 2.79 nm (Devanand and Selser, 1991). Thus, the PEGs were three orders of magnitude smaller than the granule macropores. It is therefore highly likely that the PEGs diffused completely unobstructed within the macropores. At the same time, the PEGs were only slightly smaller than the hypothesized pore size of the granule micropores. Still, the diffusion experiments indicated that the PEGs with molecular weight up to 4000 Da (1.65 nm hydrodynamic radius) penetrated throughout the entire granule, as the expected equilibrium concentration was reached. Given that the expected equilibrium was not reached with 10 kDa PEG, it is possible that this molecule was excluded from the micropores. The hydrodynamic radius of the 10 kDa PEG is 2.79 nm (Devanand and Selser, 1991), which means that the diameter of the micropores might be around 5 nm. Still, the micropores are apparently large enough to allow diffusion of PEGs up to a molecular weight of 4000 Da or a hydrodynamic radius up to 1.65 nm. The notion that macropores or channels significantly enhance diffusion into the granules is therefore incorrect for molecules lighter than 4000 Da. For these molecular weights, diffusion in the macropores is only marginally faster than diffusion in the micropores. In contrast, molecules that are excluded from the micropores, can only diffuse in the macropores. The heterogeneity of the granules is therefore mainly of relevance for heavier molecules (≥ 4 kDa).

This discussion highlights that diffusion behaviour in the granules is closely linked to the structure of the EPS matrix. Our results are therefore not universally applicable to all biofilm types. The aerobic granules used in this study have a distinct heterogeneous structure with macropores (10–20 µm in diameter) and fibrils. Biofilms with alginate as major component might have a structure more similar to alginate beads (see Fig. 3F) and the diffusive behaviour in these biofilms can be very different. It is therefore of utmost importance that studies of diffusion in biofilms describe both the diffusion behaviour and the structure of the EPS matrix.

### 4.3 Implications for practice

An important question in the design and operation of AGS systems is which fraction of influent COD can rapidly diffuse into the granules during the anaerobic phase of the sequencing batch cycle (Pronk et al., 2015). This diffusible fraction is important for granule formation and for nutrient removal (Layer et al., 2019). The lack of a significant effect of the granule matrix on the relative diffusivity D_e_/D_aq_ suggests that even larger molecules (up to 4000 Da) are likely to be available for conversion within the granules. Because of their size, larger molecules already diffuse slower in water than small molecules, but the granule matrix does not limit their diffusion further. We can evaluate the transient penetration of a molecule into a granule with the following equation (Crank, 1975):

(6) $$\frac{C}{C_{b}}=1+2\sum_{n=1}^{\infty }{\left(-1\right)}^{n}\exp\left(-\frac{D_{e}n^{2}\pi^{2}t}{R^{2}}\right)$$

Here, C is the concentration in the granule core, C_b_ is the concentration in the bulk liquid, R is the granule radius, D_e_ is the diffusion coefficient, and t is the time. This equation can be used to estimate the concentration in the granule core after a certain time period. We can estimate the diffusive penetration after 60 min, which is the length of the anaerobic feeding period for most AGS reactors (de Kreuk and Van Loosdrecht, 2004; Pronk et al., 2015). The diffusive penetration is expressed by the relative concentration in the granule core, C/C_b_. With the diffusion coefficients that were found in this study (for 62–4000 Da PEGs), granules with a 1 mm radius, and a temperature of 10 °C, the relative concentration in the granule core (C/C_b_) ranges from 54 to 100% after 60 min; all molecules of 1 kDa and lighter reach a relative concentration in the granule core of at least 93% of the concentration in the bulk. The molecules between 1 kDa and 4 kDa penetrate less and reach a C/C_b_ between 47 and 93%. Of course, the in situ penetration depth depends on both the diffusive properties as well as the consumption rate. Still, this analysis shows that for many molecules with different size, diffusion does not limit penetration depth.

The above analysis has even stronger implications if the distribution of soluble COD is considered. Of the soluble COD in the three analysed wastewater samples, 61–69% was lighter than 1 kDa and 70–87% was lighter than 10 kDa. This means that at least 61–69% of the soluble COD can diffuse easily into the granules. It is therefore likely that the majority of the soluble COD is converted within the granules, where it can contribute to nutrient removal (Layer et al., 2019, 2020). Nevertheless, the composition of wastewater can vary significantly between different locations (Henze and Harremoës, 1992). We found a similar COD distribution for three wastewaters which originated from different sewer systems in the Netherlands. Nevertheless, similar soluble COD distributions were found by other authors, for domestic, textile, tannery, and agricultural wastewaters (Doğruel, 2012; Dulekgurgen et al., 2006; Hu et al., 2002; Karahan et al., 2008; Ravndal et al., 2018). It is however not clear if the abundance of < 1 kDa COD is an innate property of wastewater or if it is the result of conversion processes within the sewer. More research is required to determine how universally applicable our findings are.

There are two practical lessons that can be learned from this study. The first lesson relates to the characterization of wastewater for AGS. The majority of the soluble COD, measured by filtration over a 0.45 µm filter, is small enough to diffuse into AGS. This means that a simple characterization approach suffices to know which fraction of influent COD can be converted within the granules. Even though not all soluble COD is truly diffusible, it is a reasonable approximation. The benefit of a full ultrafiltration characterization is small, especially considering the pitfalls of this method (Logan and Jiang, 1990). The second lesson relates to AGS models. The majority of AGS models are based on simple substrates, like VFAs (Baeten et al., 2019; Ni and Yu, 2010). The diffusivity of these substrates is generally assumed to be 80% of their diffusivity in water (Nicolella et al., 1998; Wanner et al., 2006). Our findings show that the use of a single reduction factor for diffusion in granules is valid for molecules with a molecular weight up to 4000 Da. Thus, AGS models do not require a unique reduction factor for each individual molecule. However, for a full consideration of diffusional aspects in AGS charge and hydrophobicity aspects need to be studied as well.

## 5 Conclusion

In this study, the effect of the molecular weight of a solute on its diffusion coefficient in aerobic granules was evaluated, next to the granule structure and the distribution of soluble COD in influent wastewater. There was no statistically significant difference for the diffusion coefficients in water or granules for PEGs with a molecular weight between 62 and 4000 Da. This indicates that within this molecular weight range, diffusing molecules were only marginally obstructed by the granule matrix. A 10 kDa PEG molecule was partially excluded from the granules and only accessed 65% of the granule volume. The granule structure was heterogeneous, with large macropores (∼10 µm diameter) and semi-solid regions that contained microbial cells. The partial exclusion of the 10 kDa PEG suggests that the semi-solid regions contain micropores with a diameter around 5 nm. Thus, the diffusion results provide practical information, but they also contribute to a characterization of the granule matrix.

Interestingly, a relatively large fraction (61–69%) of the soluble COD in influent wastewater is lighter than 1 kDa and thus can diffuse rapidly into the granules. These findings can be used to simplify AGS models and influent characterization approaches. AGS models do not need to consider the effect of molecular weight on the diffusion coefficients, as for most molecules the diffusion coefficient in the granule is not significantly different from its diffusion coefficient in water. The characterization of influent COD for AGS reactors can suffice with a simple filtration with a 0.45 µm pore size filter, as the majority of soluble COD in domestic wastewater is also diffusible inside aerobic granular sludge.

## Disclosure statement

There are no conflicts of interest to declare.

## Declaration of Competing Interest

The authors declare that they have no known competing financial interests or personal relationships that could have appeared to influence the work reported in this paper.

## Appendix Supplementary materials

Image, application 1
