# Supplementary material for: Diffusion of soluble organic substrates in aerobic granular sludge: Effect of molecular weight
Source: Water Res X. 2022 Jul 2;16:100148. doi: 10.1016/j.wroa.2022.100148 (PMC9263526; doi:10.1016/j.wroa.2022.100148)
Supplement: Supplementary file 1 [file mmc1.docx]

**Supplementary information**

**Diffusion of Soluble Organic Substrates in Aerobic Granular Sludge: Effect of Molecular Weight**

Lenno van den Berg^1*^, S. Toja Ortega^1^, Mark C.M. van Loosdrecht^2^, Merle K. de Kreuk^1^

^1^ Department of Water Management, Delft University of Technology, The Netherlands

^2^ Department of Biotechnology, Delft University of Technology, The Netherlands

*Corresponding author. Stevinweg 1, 2628 CN Delft, The Netherlands. E-mail: [L.vandenBerg@tudelft.nl](mailto:L.vandenBerg@tudelft.nl)

**Table A.1. Diffusion experiment data, including granule properties that have been measured as part of the diffusion experiment. Here, D_aq_ is the diffusion coefficient of a molecule in water and D_e_ is the diffusion coefficient of a molecule within the granule. The D_e_ at 4 °C is the outcome of the fitting procedure, and the D_e_ at 25 °C is recalculated based on Einstein’s equation. All values are given as mean, with or without standard deviation.**

| **Solute** | **Volume** | **Radius** | **Aspect ratio** | **Buoyant density** | **Biomass density** | **Ash** | **D_aq_** | **D_e_ (4 °C)** | **D_e_ (25 °C)** | **D_e_/D_aq_** |
| --- | --- | --- | --- | --- | --- | --- | --- | --- | --- | --- |
|  | **mL** | **mm** | **-** | **kg/m^3^** | **gVSS/L** | **%** | **x 10^-10^ m^2^/s** | **x 10^-10^ m^2^/s** | **x 10^-10^ m^2^/s** | **-** |
| PEG62 | 60.3 ± 0.5 | 1.43 ± 0.24 | 1.47 ± 0.58 | 1019.4 | 57.5 | 21.8 | 9.51 | 4.25 ± 1.38 | 7.95 ± 2.58 | 0.84 ± 0.27 |
| PEG106 | 52.7 ± 0.8 | 1.44 ± 0.20 | 1.44 ± 0.47 | 1011.7 | 54.8 | 21.0 | 6.91 | 2.70 ± 0.82 | 5.05 ± 1.53 | 0.73 ± 0.22 |
| PEG200 | 56.2 ± 1.2 | 1.50 ± 0.23 | 1.42 ± 0.44 | 1017.6 | 62.6 | 19.4 | 4.73 | 3.08 ± 1.01 | 5.75 ± 1.89 | 1.22 ± 0.40 |
| PEG300 | 60.0 ± 0.6 | 1.50 ± 0.23 | 1.42 ± 0.44 | 1020.0 | 58.9 | 21.3 | 3.71 | 1.63 ± 0.51 | 3.05 ± 0.96 | 0.82 ± 0.26 |
| PEG400 | 64.6 ± 0.9 | 1.50 ± 0.23 | 1.42 ± 0.44 | 1018.8 | 59.5 | 20.9 | 3.12 | 1.72 ± 0.55 | 3.21 ± 1.03 | 1.03 ± 0.33 |
| PEG600 | 58.1 ± 0.6 | 1.50 ± 0.23 | 1.42 ± 0.44 | 1024.2 | 58.0 | 21.0 | 2.45 | 1.15 ± 0.37 | 2.15 ± 0.68 | 0.88 ± 0.28 |
| PEG1000 | 59.9 ± 0.6 | 1.44 ± 0.20 | 1.44 ± 0.47 | 1017.9 | 53.8 | 20.9 | 1.81 | 1.07 ± 0.30 | 2.00 ± 0.56 | 1.11 ± 0.31 |
| PEG1500 | 64.0 ± 0.8 | 1.43 ± 0.24 | 1.47 ± 0.58 | 1021.2 | 57.8 | 21.7 | 1.42 | 0.87 ± 0.30 | 1.63 ± 0.56 | 1.15 ± 0.40 |
| PEG2000 | 55.4 ± 0.7 | 1.43 ± 0.24 | 1.47 ± 0.58 | 1017.5 | 56.1 | 21.4 | 1.19 | 0.50 ± 0.17 | 0.94 ± 0.31 | 0.79 ± 0.26 |
| PEG3000 | 65.8 ± 0.6 | 1.44 ± 0.20 | 1.44 ± 0.47 | 1023.4 | 60.7 | 19.5 | 0.94 | 0.39 ± 0.11 | 0.73 ± 0.21 | 0.78 ± 0.22 |
| PEG4000 | 66.7 ± 0.3 | 1.44 ± 0.20 | 1.44 ± 0.47 | 1019.7 | 58.4 | 20.2 | 0.79 | 0.49 ± 0.14 | 0.91 ± 0.27 | 1.16 ± 0.34 |
| PEG10000 | 65.3 ± 0.8 | 1.35 ± 0.24 | 1.43 ± 0.43 | 1019.8 | 63.3 | 19.8 | 0.46 | - | - | - |

| 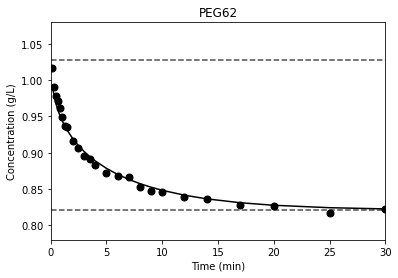 | 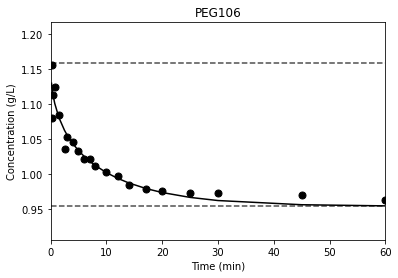 | 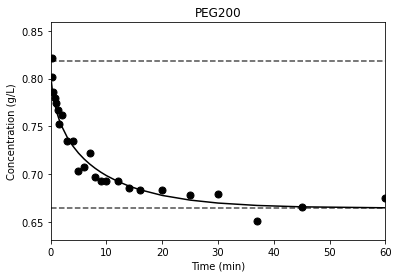 | 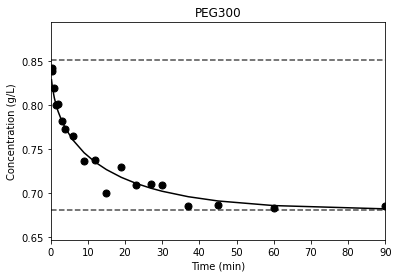 |
| --- | --- | --- | --- |
| 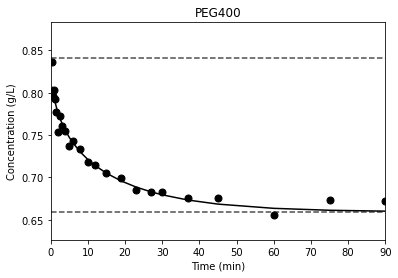 | 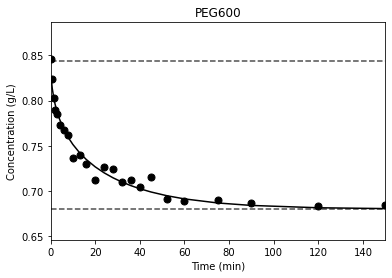 | 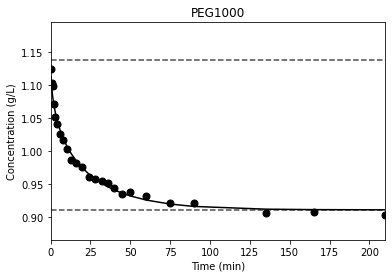 | 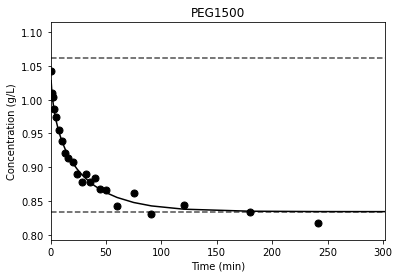 |
| 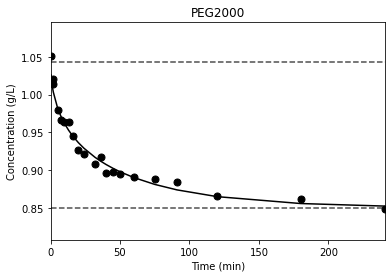 | 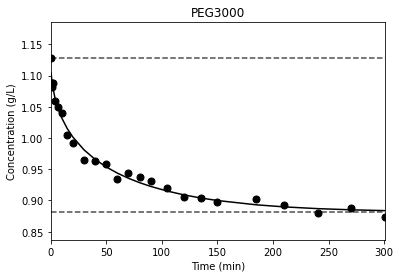 | 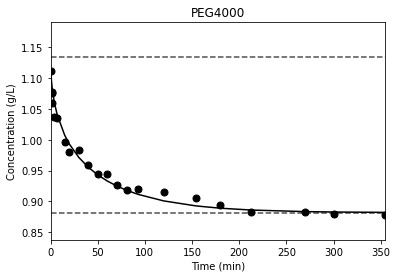 | 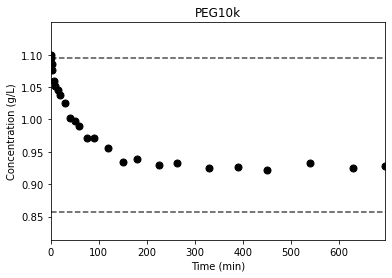 |
| **Figure A.1. Concentration profiles during the diffusion experiment and fitted diffusion coefficient.** | | | |

**Table A.2. Overview of the studies included in the review of Stewart (1998) and their major limitations.**

|  | **Reference** | **Steady-state reaction method** | **High biofilm density** | **Diffusion in compressed flocs** | **Reaction not considered** |
| --- | --- | --- | --- | --- | --- |
| 1 | Fan, Leyva‐Ramos, Wisecarver, and Zehner (1990) |  | X |  |  |
| 2 | Onuma and Omura (1982) |  |  | X |  |
| 3 | Pipes, Characklis, and Matson (1974) as cited in Fan et al. (1990) |  |  | X |  |
| 4 | Matson and Characklis (1976) |  |  | X |  |
| 5 | Dibdin (1981) |  | X |  |  |
| 6 | la Cour Jansen and Harremoes (1985) | X |  |  |  |
| 7 | Dibdin (1993) |  | X |  |  |
| 8 | McNee, Geddes, and Weetman (1982) |  | X |  |  |
| 9 | Beyenal and Tanyolac (1994) | X |  |  |  |
| 10 | Andrews and Tien (1981) | X |  |  |  |
| 11 | Baillod and Boyle (1970) |  | X | X |  |
| 12 | Fujie, Tsukamoto, and Kubota (1979) as cited in (Fan et al., 1990) | X |  |  |  |
| 13 | LaMotta (1976b) | X |  |  |  |
| 14 | LaMotta (1976a) | X |  |  |  |
| 15 | Livingston and Chase (1989) | X |  | X |  |
| 16 | Ozturk, Palsson, and Thiele (1989) | X |  | X |  |
| 17 | Tang and Fan (1987) | X |  |  |  |
| 18 | Tatevossian (1979) |  |  |  | X |
| 19 | Wang and Tien (1984) | X |  |  |  |
| 20 | Yu and Pinder (1993) | X |  |  |  |
| 21 | Yu and Pinder (1994) | X |  |  |  |

**References**

Andrews, G. F., & Tien, C. (1981). Bacterial Film Growth in Adsorbent Surfaces. *AlChE Journal, 27*, 396-403. doi:10.1002/aic.690270309

Baillod, C. R., & Boyle, W. C. (1970). Mass transfer limitations in substrate removal. *Journal of the Sanitary Engineering Division, 96*(2), 525-545. doi:10.1061/JSEDAI.0001092

Beyenal, H., & Tanyolac, A. (1994). The calculation of simultaneous effective diffusion coefficients of the substrates in a fluidized bed biofilm reactor. *Water Science and Technology, 29*(10-11), 463. doi:10.2166/wst.1994.0793

Dibdin, G. (1981). Diffusion of sugars and carboxylic acids through human dental plaque in vitro. *Archives of oral biology, 26*(6), 515-523. doi:10.1016/0003-9969(81)90010-8

Dibdin, G. (1993). Effect of the bathing fluid on measurements of diffusion in dental plaque. *Archives of oral biology, 38*(3), 251-254. doi:10.1016/0003-9969(93)90035-K

Fan, L. S., Leyva‐Ramos, R., Wisecarver, K., & Zehner, B. (1990). Diffusion of phenol through a biofilm grown on activated carbon particles in a draft‐tube three‐phase fluidized‐bed bioreactor. *Biotechnology and Bioengineering, 35*(3), 279-286. doi:10.1002/bit.260350309

Fujie, K., Tsukamoto, T., & Kubota, H. (1979). Reaction kinetics of wastewater treatment with a microbial film. *J. Ferment. Technol., 57*, 539-545.

la Cour Jansen, J., & Harremoes, P. (1985). Removal of soluble substrates in fixed films. *Water Science & Technology, 17*(2-3), 1-14. doi:10.2166/wst.1985.0115

LaMotta, E. J. (1976a). External mass transfer in a biological film reactor. *Biotechnology and Bioengineering, 18*(10), 1359-1370. doi:10.1002/bit.260181004

LaMotta, E. J. (1976b). Internal diffusion and reaction in biological films. *Environmental Science & Technology, 10*(8), 765-769. doi:10.1021/es60119a003

Livingston, A. G., & Chase, H. A. (1989). Modeling phenol degradation in a fluidized‐bed bioreactor. *AIChE journal, 35*(12), 1980-1992. doi:10.1002/aic.690351209

Matson, J. V., & Characklis, W. G. (1976). Diffusion into microbial aggregates. *Water Research, 10*, 877-885. doi:10.1016/0043-1354(76)90022-1

McNee, S., Geddes, D., & Weetman, D. (1982). Diffusion of sugars and acids in human dental plaque in vitro. *Archives of oral biology, 27*(11), 975-979. doi:10.1016/0003-9969(82)90106-6

Onuma, M., & Omura, T. (1982). Mass-transfer characteristics within microbial systems. *Water Science & Technology, 14*(6-7), 553-568. doi:10.2166/wst.1982.0125

Ozturk, S. S., Palsson, B. O., & Thiele, J. H. (1989). Control of interspecies electron transfer flow during anaerobic digestion: dynamic diffusion reaction models for hydrogen gas transfer in microbial flocs. *Biotechnology and Bioengineering, 33*(6), 745-757. doi:10.1002/bit.260330612

Pipes, D. M., Characklis, W. G., & Matson, J. V. (1974). Discussion of “Substrate Removal Mechanism of Trickling Filters”. *Journal of the Environmental Engineering Division, 100*(1), 225-226. doi:10.1061/JEEGAV.0000149

Stewart, P. S. (1998). A review of experimental measurements of effective diffusive permeabilities and effective diffusion coefficients in biofilms. *Biotechnology and Bioengineering, 59*, 261-272. doi:10.1002/(SICI)1097-0290(19980805)59:3<261::AID-BIT1>3.0.CO;2-9

Tang, W. T., & Fan, L. S. (1987). Steady state phenol degradation in a draft‐tube, gas‐liquid‐solid fluidized‐bed bioreactor. *AlChE Journal, 33*(2), 239-249. doi:10.1002/aic.690330210

Tatevossian, A. (1979). Diffusion of radio tracers in human dental plaque. *Carries research, 13*(3), 154-162. doi:10.1159/000260396

Wang, S. C. P., & Tien, C. (1984). Bilayer film model for the interaction between adsorption and bacterial activity in granular activated carbon columns part I: Formulation of equations and their numerical solutions. *AlChE Journal, 30*(5), 786-794. doi:10.1002/aic.690300513

Yu, J., & Pinder, K. (1993). Diffusion of lactose in acidogenic biofilms. *Biotechnology and Bioengineering, 41*(7), 736-744. doi:10.1002/bit.260410708

Yu, J., & Pinder, K. L. (1994). Effective diffusivities of volatile fatty acids in methanogenic biofilms. *Bioresource Technology, 48*(2), 155-161. doi:10.1016/0960-8524(94)90203-8
